# Bioactivity and Structural Properties of Novel Synthetic Analogues of the Protozoan Toxin Climacostol

**DOI:** 10.3390/toxins11010042

**Published:** 2019-01-15

**Authors:** Federico Buonanno, Elisabetta Catalani, Davide Cervia, Francesca Proietti Serafini, Simona Picchietti, Anna Maria Fausto, Simone Giorgi, Gabriele Lupidi, Federico Vittorio Rossi, Enrico Marcantoni, Dezemona Petrelli, Claudio Ortenzi

**Affiliations:** 1 Laboratory of Protistology and Biology Education, Department of Education, Cultural Heritage, and Tourism (ECHT), Università degli Studi di Macerata, 62100 Macerata, Italy; federico.buonanno@unimc.it; 2 Department for Innovation in Biological, Agro-food and Forest systems (DIBAF), Università degli Studi della Tuscia, 01100 Viterbo, Italy; ecatalani@unitus.it (E.C.); d.cervia@unitus.it (D.C.); pserafini@unitus.it (F.P.S.); picchietti@unitus.it (S.P.); fausto@unitus.it (A.M.F.); 3 School of Sciences and Technologies, Section of Chemistry, Università degli Studi di Camerino, 62032 Camerino, Italy; simone.giorgi@unicam.it (S.G.); gabriele.lupidi@unicam.it (G.L.); federico.rossi@unicam.it (F.V.R.); enrico.marcantoni@unicam.it (E.M.); 4 School of Biosciences and Veterinary Medicine, Università degli Studi di Camerino, 62032 Camerino, Italy; dezemona.petrelli@unicam.it

**Keywords:** *Climacostomum virens*, secondary metabolites, resorcinolic lipids, protists, ciliates, apoptosis

## Abstract

Climacostol (5-[(2*Z*)-non-2-en-1-yl]benzene-1,3-diol) is a resorcinol produced by the protozoan *Climacostomum virens* for defence against predators. It exerts a potent antimicrobial activity against bacterial and fungal pathogens, inhibits the growth of several human and rodent tumour cells, and is now available by chemical synthesis. In this study, we chemically synthesized two novel analogues of climacostol, namely, 2-methyl-5 [(2*Z*)-non-2-en-1-yl]benzene-1,3-diol (AN1) and 5-[(2*Z*)-non-2-en-1-yl]benzene-1,2,3-triol (AN2), with the aim to increase the activity of the native toxin, evaluating their effects on prokaryotic and free-living protists and on mammalian tumour cells. The results demonstrated that the analogue bearing a methyl group (AN1) in the aromatic ring exhibited appreciably higher toxicity against pathogen microbes and protists than climacostol. On the other hand, the analogue bearing an additional hydroxyl group (AN2) in the aromatic ring revealed its ability to induce programmed cell death in protistan cells. Overall, the data collected demonstrate that the introduction of a methyl or a hydroxyl moiety to the aromatic ring of climacostol can effectively modulate its potency and its mechanism of action.

## 1. Introduction

Natural products possess enormous structural and chemical diversity that is unsurpassed by any synthetic libraries and have provided the basis for several effective new drugs [1]. The quality of lead compounds arising from natural sources is high, due to their coevolution with the targets in biological systems. Currently, a great deal of effort is still aimed at discovering novel small molecules or trying to understand the structure–activity relationships of lead compounds. This may help to find new derivatives exhibiting the optimum in potency/selectivity, and to exploit new therapeutic indications.

Climacostol (5-[(2*Z*)-non-2-en-1-yl]benzene-1,3-diol) is a resorcinolic lipid physiologically produced by the freshwater ciliated protozoan *Climacostomum virens* for chemical defence against unicellular and multicellular predators [2]. It was structurally characterized and synthetized by Masaki et al. [3], and has more recently been obtained as a pure compound in the natural and most bioactive *Z*-configuration by a novel and straightforward synthesis [4].

In the last ten years, the studies performed to evaluate the effects of climacostol on bacteria, fungi, protozoa, human and rodent cell lines, and isolated mitochondria have indicated that the toxin exerts antimicrobial, cytotoxic, pro-apoptotic, and genotoxic activities, and that it also induces dysfunctional autophagy. These results have indicated some peculiar structural and functional traits of the molecule, among which the capability to inhibit the respiratory chain complex I in rat liver mitochondria, and the activation of the transcription factor p53 system as the molecular crossroad regulating both the anti-autophagic action of climacostol and its role in the apoptosis induction in tumours [4,5,6,7,8,9,10,11].

Like all resorcinolic lipids, climacostol has a dual, hydrophilic and hydrophobic character, due to the presence in its molecule of a hydroxylated aromatic ring and a straight hydrocarbon chain. Apart from simple 5-alkylresorcinols isolated from plants and bacteria, the occurrence of various derivatives (ring- or chain-modified) has also been demonstrated [12]. For example, the side chain of these compounds can be saturated or it can carry one to four double bonds in a *cis* configuration, with the localization of double bonds usually related to the chain length [13].

With regard to the hydrocarbon chain of climacostol, Buonanno and Ortenzi [6] demonstrated that the compound’s cytotoxic potency on a panel of various species of free-living freshwater ciliated protozoa can be modulated by the substitution of the double bond with a single or a triple one. This was easily achieved thanks to the availability of synthetic preparations of alkyl and alkynyl derivatives of climacostol [14]. The data collected demonstrated that the cytotoxic potential of climacostol and its two derivatives was directly related to the saturation rate of the hydrocarbon chain, with the native compound showing intermediate activity. Unlike what has been reported for protozoa and microbes, the aromatic ring of climacostol was supposed to play a key role in the inhibition of the growth of mammalian tumour cells [9]. In particular, an apoptotic process is triggered after DNA damage that follows the generation of reactive oxygen species (ROS) in the presence of Cu(II). Of interest, climacostol triggers the death process of tumour cells as a result of early DNA binding and damage [10]. In this respect, in vitro and in vivo results indicated that climacostol exerts an effective and potent cytotoxic, pro-apoptotic, and anti-autophagic action on multiple tumour cells, including the inhibition of mouse melanoma progression thus increasing animal survival [1,10,11]. This evidence indicates climacostol as a highly effective compound to be considered for the design of new anti-cancer drugs.

Anti-cancer properties were also recognized for some polyphenols that can mobilize endogenous copper in human peripheral lymphocytes leading to oxidative DNA breakage [15]. Among these, resveratrol, a natural phenol extracted from grapes, nuts, and other plants, and sharing with climacostol the same hydroxylated aromatic ring, was also reported to induce DNA damage in human lymphocytes in the presence of cupric ions [16]. Interestingly, it was demonstrated that the addition of a hydroxyl group to the *ortho* position of the aromatic ring contributes to significantly increasing the cytotoxic activity of resveratrol against HL-60 cancer cells [17]. On the other hand, the presence of a methyl group in the hydroxylated aromatic ring of phenolic lipids can significantly enhance their antimicrobial activity. This is the case with 5-methylresorcinols isolated from corn pericarp wax that protects the kernel against *Aspergillus flavus* infection and aflatoxin production [11,18]; in addition, 5-methylresorcinols isolated from *Pseudomonas* sp. Ki19 can inhibit the growth of fungi and Gram-positive bacteria such as *Aspergillus fumigatus*, *Fusarium culmorum*, and *Staphylococcus aureus* [19].

**Figure 1:**
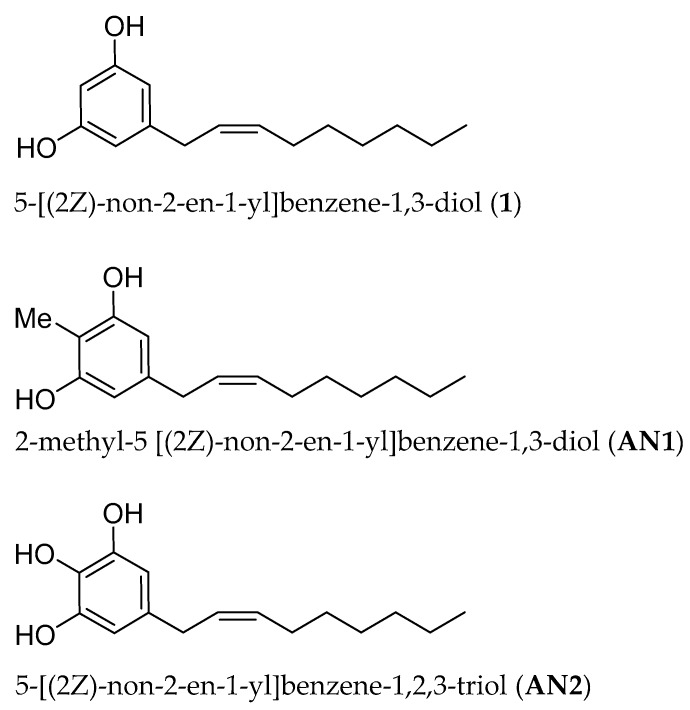
Molecular structures of climacostol (**1**), **AN1**, and **AN2**.

These observations led us to study two new synthetic analogues of climacostol, namely, 2-methyl-5 [(2*Z*)-non-2-en-1-yl]benzene-1,3-diol (AN1) and 5-[(2*Z*)-non-2-en-1-yl]benzene-1,2,3-triol (AN2), carrying an additional methyl group and a hydroxyl group, respectively, in the aromatic ring (Figure 1). In fact, in the course of researching the structure–activity relationship of climacostol and the mechanism of its pro-oxidant effect, an initial hydroxylation of the aromatic ring was observed [5]. Among the analogues of climacostol, we therefore selected AN1 and AN2 because (i) they do not violate Lipinski’s rule of five, (ii) in DNA cleavage mediated by climacostol the oxygenation required in the aromatic ring does not involve the 2-position, and (iii) a trihydroxylated species acts as an intermediary in the initial hydroxylation of the aromatic ring of alkenyl resorcinol such as climacostol. Compounds were tested for their biological effects on mammalian cells, and on pathogenic microbes and free-living ciliated protists previously studied for their sensitivity to alkyl and alkynyl derivatives of climacostol [6,8], in order to better understand the structure–activity relationships.

The main aim of this study was to identify the structural traits of the native climacostol that could be modified to increase its anti-tumour and/or antimicrobial activities.

## 2. Results

### 2.1. Synthesis of Climacostol and Its Analogues

Climacostol (5-[(2*Z*)-non-2-en-1-yl]benzene-1,3-diol) was synthesized as described in Reference [4].

**Figure 2:**
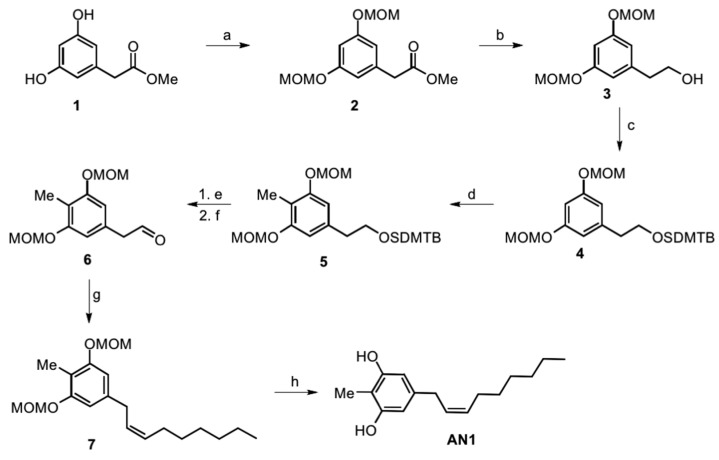
Total synthesis of the AN1 analogue: a) MOM-Cl, DIPEA, DCM, 0 °C to r.t., o.n., 74%; b) LiAlH_4_, THF, 0 °C, 94%; c) DIPEA, TBDMSCl, DMF, o.n., 96%; d) BuLi, THF, −78 °C, 1 h, then MeI, −78 °C to r.t., o.n., 75%; e) TBAF, THF, r.t., o.n., 96%; f) DMP, DCM, 0 °C to r.t., o.n., 80%; g) anhydrous CeCl_3_, THF dry, 2 h, then added dropwise solution of *n*-heptyltriphenylphosphonium bromide and NaHMDS in THF dry, o.n., 0 °C, 83%; h) *p*-TosOH·H_2_O, MeOH:DCM 1:1, r.t., o.n., 98%.

For the methyl-substituted climacostol analogue AN1 (2-methyl-5 [(2*Z*)-non-2-en-1-yl]benzene-1,3-diol), no starting material with all the phenyl ring substituents was available, and we therefore started from the same starting material as the climacostol synthesis. We performed (Figure 2) the directed *ortho*-methylation (DOM) to introduce the methyl group, and this reaction is favoured from the MOM-protecting groups in intermediate 4 which have a strong directing effect on the *ortho* position [20]. The DOM reaction was carried out using n-BuLi with MeI quench, and for this we had to avoid alkyllithium-reactive ester by reducing the ester moiety to the corresponding alcohol and its protection with the *tert*-butyldimethylsilyl group (TBDMS) [21]. After ring functionalization, deprotection with TBAF in dry THF and Dess–Martin oxidation gave the desired aldehyde 6 with an excellent yield.

Similarly to what is done in the diastereoselective synthesis of climacostol, the Wittig reaction in the presence of a Lewis acid such as CeCl_3_ provides the desired (*Z*)-alkenyl derivative 7 without the presence of (*E*)-diastereomer. The final MOM-deprotection led to the desired AN1 analogue with free hydroxy groups without olefin isomerization and in excellent yield.

**Figure 3:**
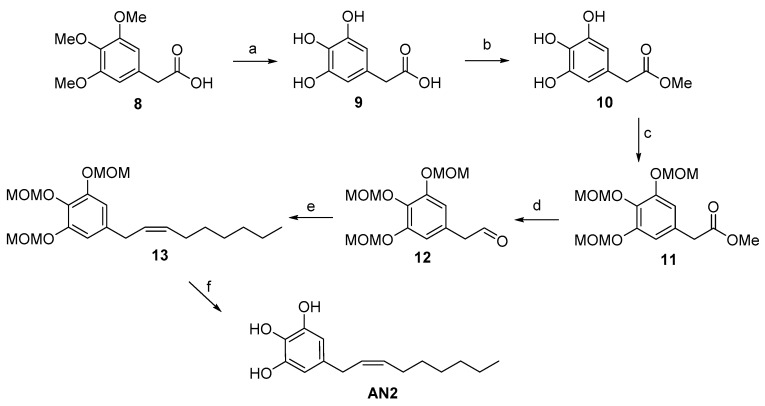
Total synthesis of the AN2 analogue: a) HBr conc., CH_3_COOH glacial, reflux, 15 h, 90%; b) Amberlyst 15, MeOH, reflux, 99%; c) MOM-Cl, DIPEA, DCM, 0 °C to r.t., o.n., 66%; d) DIBAL-H, Toluene, −78 °C, 78%; e) anhydrous CeCl_3_, THF dry, then added dropwise to a solution of *n*-heptyltriphenylphosphonium bromide, NaHMDS in THF, o.n., 0 °C, 80%; f) *p*-TosOH·H_2_O, DCM:MeOH 1:1, r.t., o.n., 98%.

With regard to the preparation of the AN2 analogue (5-[(2*Z*)-non-2-en-1-yl]benzene-1,2,3-triol) (Figure 3), the synthetic strategy started with the successful deprotection of the methyl ethers of commercially available arylacetic acid derivative 8. Our procedure allowed us to obtain intermediate 9 with a yield of 90%, unlike the 31% yield reported in literature [22]. The esterification with Amberlyst 15 allowed us to obtain the corresponding methyl ester 10, which was used for the next reaction step without further purification [23]. The subsequent treatment of intermediate 10 with an excess of chloromethyl methyl ether afforded the MOM-protected 3,4,5-trihydroxyphenylacetate 11, which was reduced to corresponding aldehyde 12 with DIBAL-H as reported in our climacostol synthesis. Wittig reaction in the presence of anhydrous CeCl_3_ led exclusively to the *Z*-isomer, and the final deprotection provided the desired compound AN2 in excellent yield, and above all, spectroscopically pure, avoiding the difficult purification on silica gel column owing to the polar nature of these compounds.

### 2.2. Spectroscopic Analysis of Climacostol and the AN1 and AN2 Analogues

**Figure 4:**
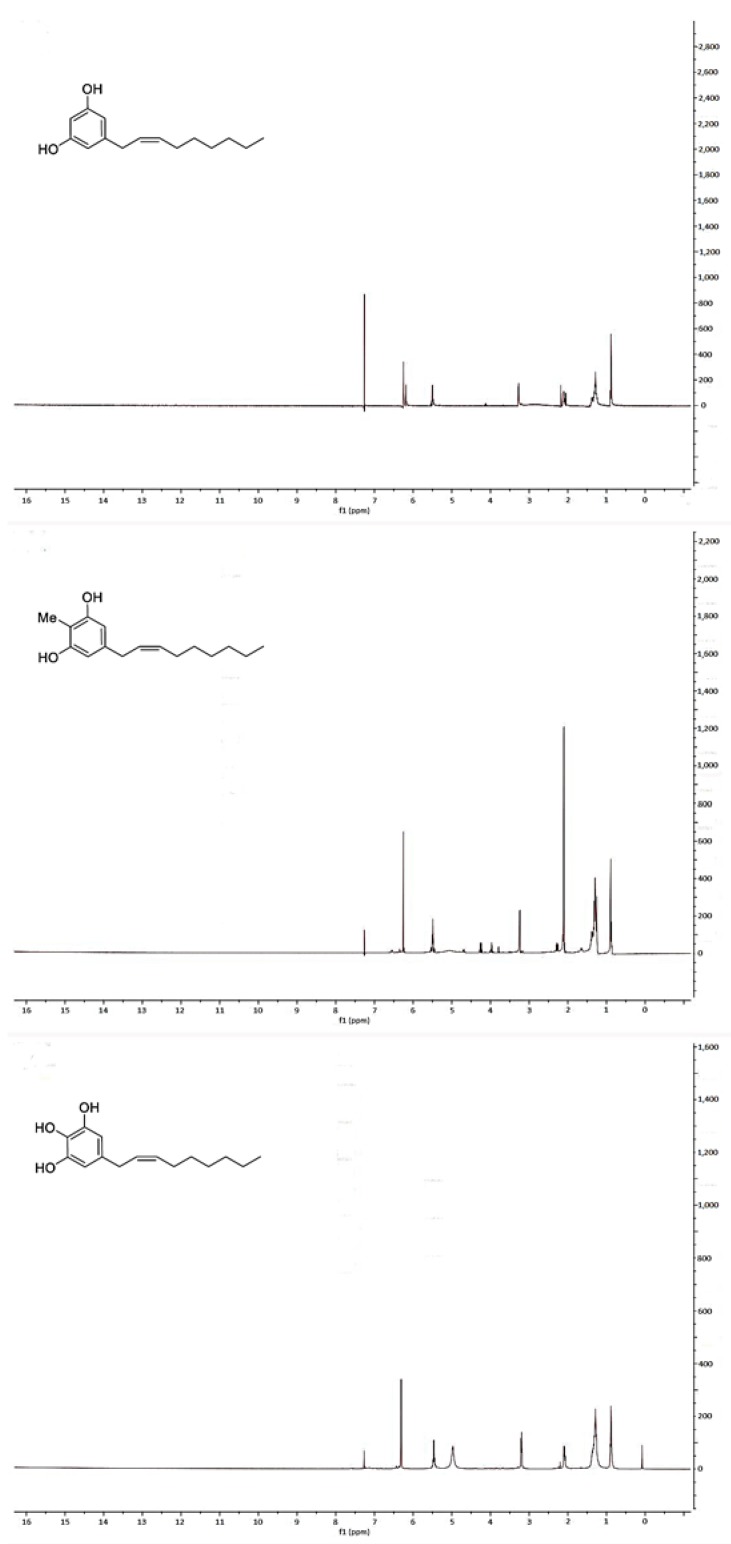
^1^H-NMR spectra of (top) climacostol, (middle) AN1, and (bottom) AN2.

**Figure 5:**
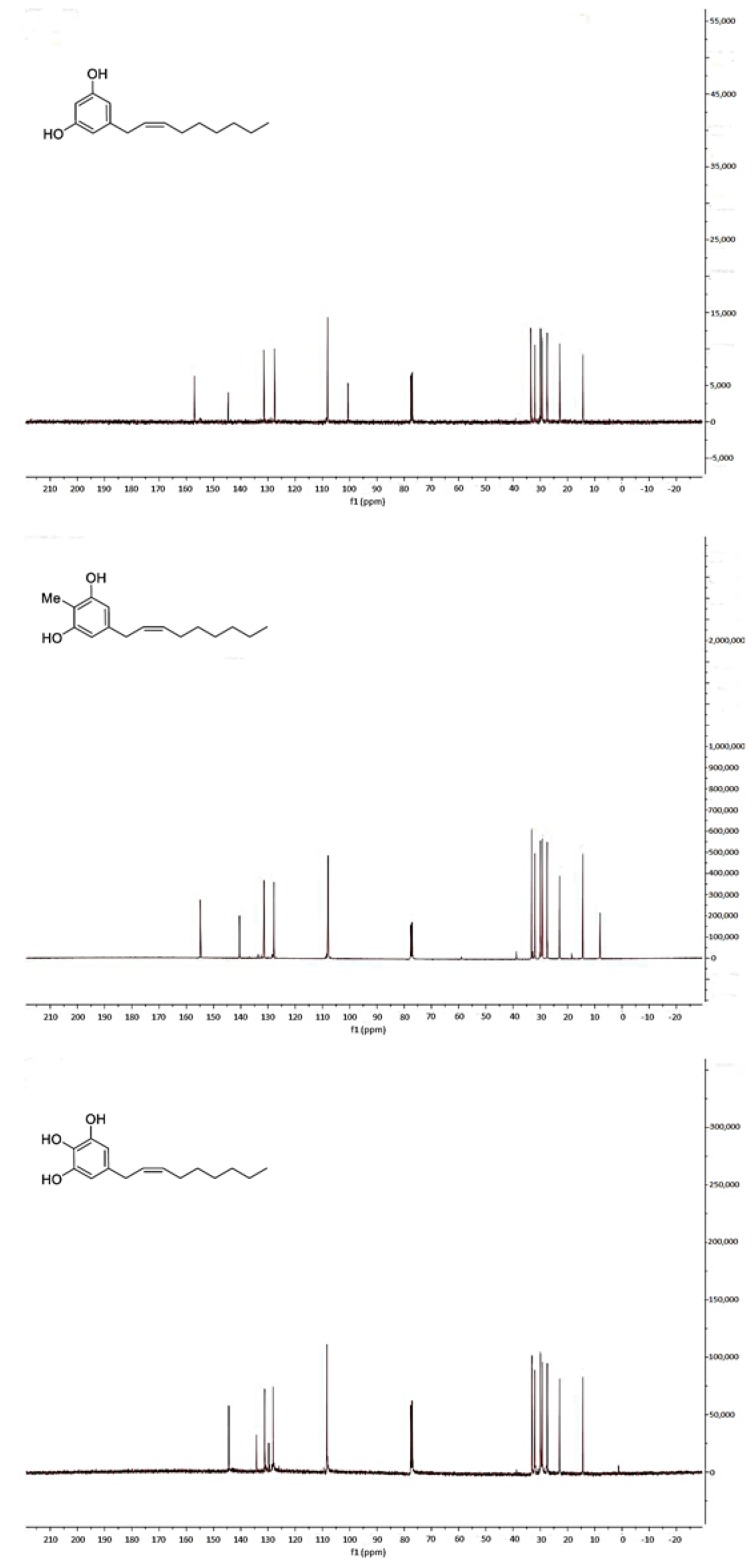
^13^C-NMR spectra of (top) climacostol, (middle) AN1, and (bottom) AN2.

All NMR spectra were acquired using a Varian 400 spectrometer (Varian – acquired Agilent Technologies, Palo Alto, CA, USA) using standard NMR tubes at 298 K, and operating at 400 MHz for ^1^H and at 100 MHz for ^13^C. Residual protic solvent CHCl_3_ (δ_H_ = 7.26) was used as the internal reference, and ^13^C NMR spectra were recorded using the central resonance of CDCl_3_ (δ_C_ = 77.0) as the internal reference. Unfortunately, it was not possible for us to confirm the stereochemistry at C-2′ and C-3′ double bond in climacostol, AN1, and AN2 (Figure 4 and Figure 5). In particular, ^1^H NMR spectrum showed that the proton signals H-2′ and H-3′ were not well separated. This meant the impossibility of using conventional 2D NMR methods such as COSY and NOESY because it is known that they fail when key protons signals are poorly resolved [24].

**Figure 6:**
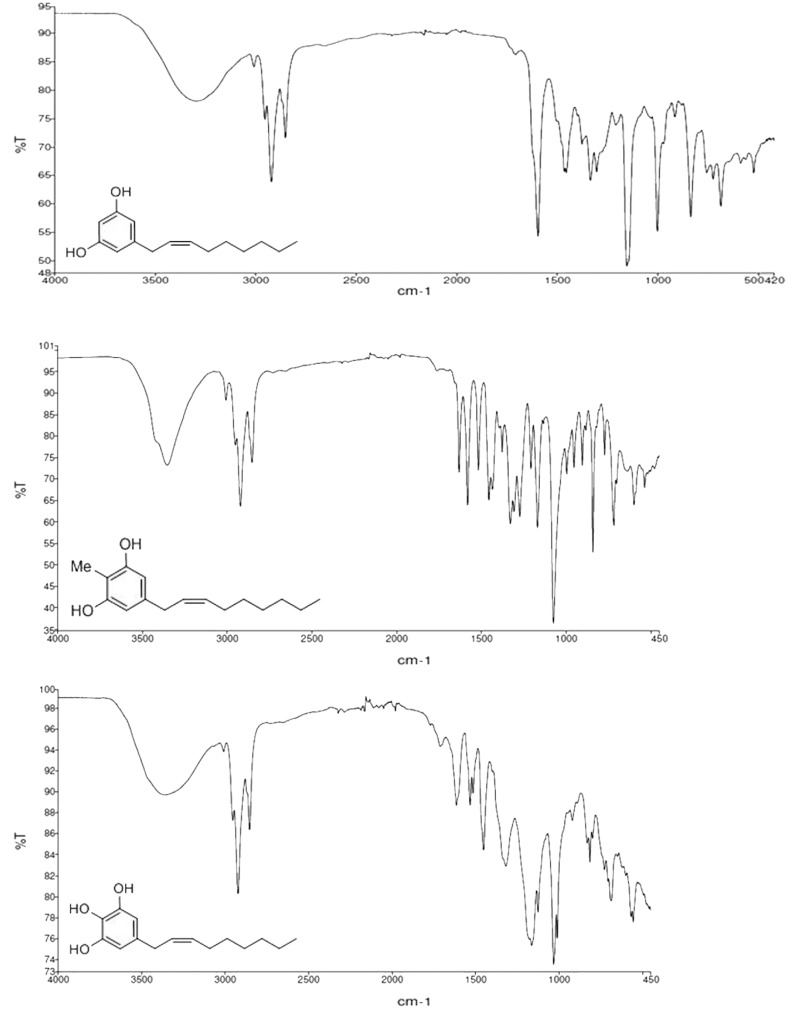
FTIR spectra of (top) climacostol, (middle) AN1, and (bottom) AN2.

We obtained more information from IR spectrum (Figure 6). IR spectra were recorded on a PerkinElmer FTIR Paragon 500 spectrometer (PerkinElmer, Waltham, MA, USA) using thin films on NaCl plates. The absence of the strong 970 cm^−1^ band for the *E*-geometry suggested the *Z*-geometry for the disubstituted double bond [25].

**Figure 7:**
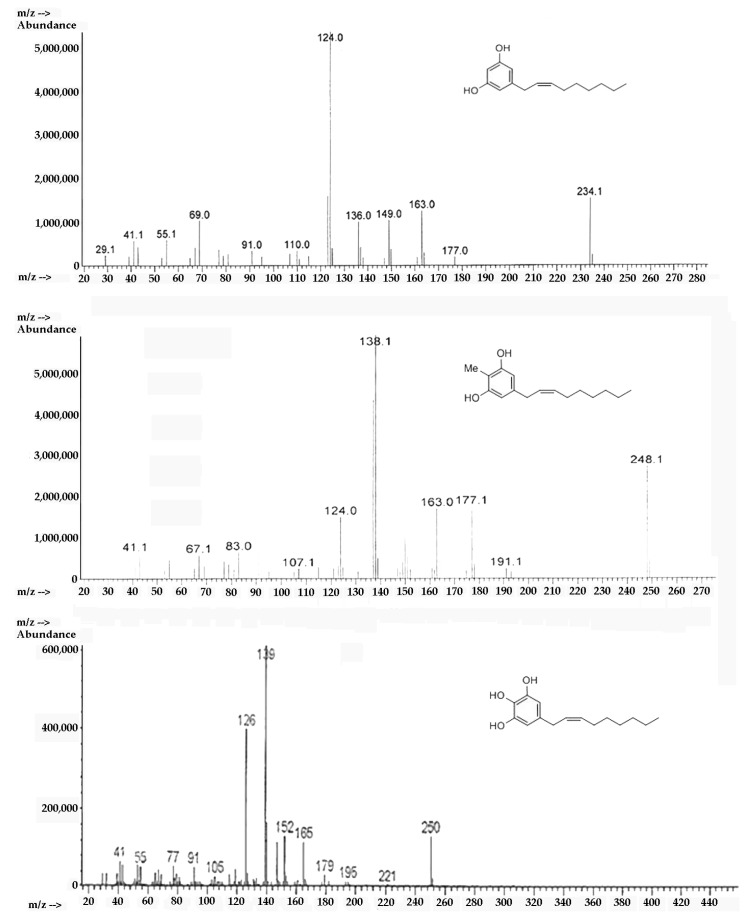
EI-MS spectra of (top) climacostol, (middle) AN1, and (bottom) AN2.

Through mass spectrometry it was possible to confirm the structure of climacostol, AN1, and AN2 in an unambiguous way. Mass spectra were recorded on an Agilent 5988 gas-chromatograph (Agilent Technologies, Santa Clara, CA, USA) with a mass-selective detector MSD 5790MS (Agilent Technologies, Santa Clara, CA, USA), utilizing electron ionization (EI) at an ionizing energy of 70 eV. A fused silica column (30 m × 0.25 mm HP-5; cross-linked 5% PhMe siloxane, 0.10 μm film thickness) was used with helium carrier flow of 30 mL/min. The temperature of the column was varied, after a delay of 3 min from the injection, from 65 to 300 °C with a slope of 15 °C/min. A characteristic electron ionization mass spectrum was observed for climacostol, AN1, and AN2 (Figure 7). The presence of notable molecular ions at *m/z* 234, *m/z* 248, and *m/z* 250, with base fragments at *m/z* 124, *m/z* 138, and *m/z* 150, respectively, due to McLafferty rearrangement of the phenolic rings, are in agreement with the proposed structures. In this stereochemical study, the presence of odd-electron ions *m/z* 124, *m/z* 138, and *m/z* 150 in conjunction with *m/z* 137, *m/z* 151, and *m/z* 163, respectively, was even more diagnostic. These data confirmed the presence of a double bond between carbons C-2′ and C-3′ [26], and in our procedures the isomerization of the carbon–carbon double bond (Δ^2′,3′^-Δ^1′,2′^) was not observed.

### 2.3. Detrimental Effects of Climacostol Analogues on Mammalian Cells

**Figure 8:**
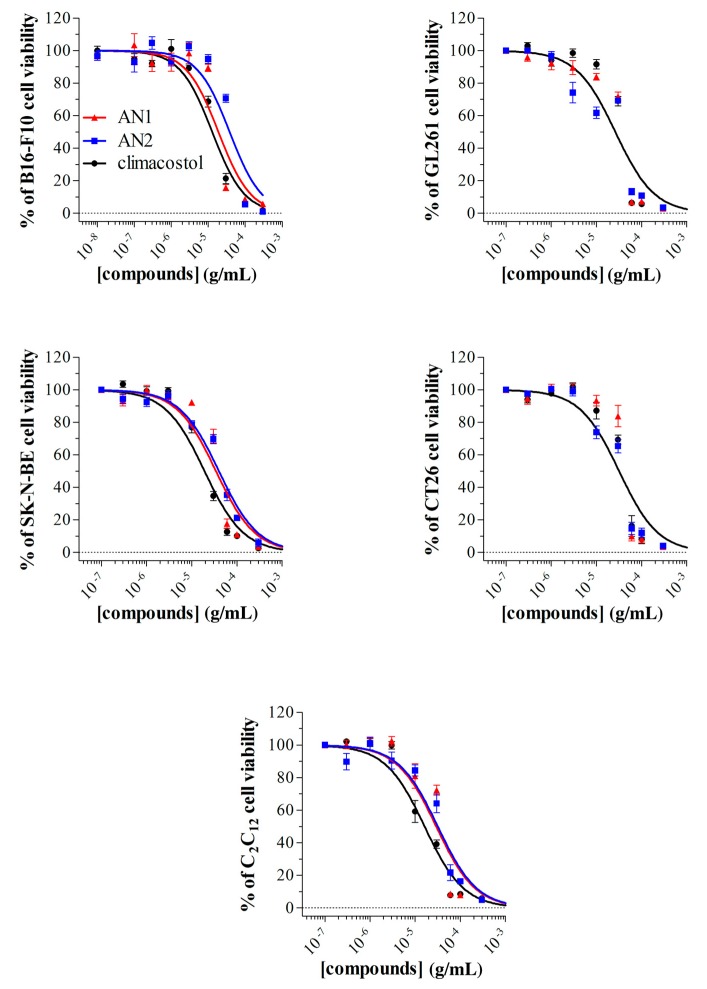
Cytotoxic properties on mammalian cells. The viability of B16-F10, GL261, SK-N-BE, CT26, and C_2_C_12_ cells treated with increasing concentrations of AN1, AN2, and climacostol for 24 h was assessed by MTT assay. Data are expressed by setting the absorbance of the reduced MTT in the absence of compounds as 100%. The data points represent the mean ± SEM of results obtained from 4–6 independent experiments.

We have recently described the cytotoxic and pro-apoptotic activity of the native molecule climacostol in mammalian cells [1,10,11]. For the sake of comparison, we now tested the in vitro cytotoxic properties of AN1 and AN2 on different immortalised cells of both tumour (B16-F10, GL261, SK-N-BE, and CT26 cells) and non-tumour (C_2_C_12_ cells) origin, by MTT assay. Cell treatment with AN1, AN2, and climacostol (24 h) caused a concentration-dependent reduction of MTT absorbance with an E_max_ concentration value of approximately 30 μg/mL (Figure 8). At the E_max_ concentration, compounds decreased cell viability by between 90% and 100%. Data also indicated that the viability of cells was negatively affected by AN1 and AN2 with a comparable potency, that is, EC_50_ ranging from approximately 18 to 40 μg/mL (Table 1). Noteworthy is that both AN1 and AN2 displayed similar or even lower potencies when compared to climacostol.

**Figure 9:**
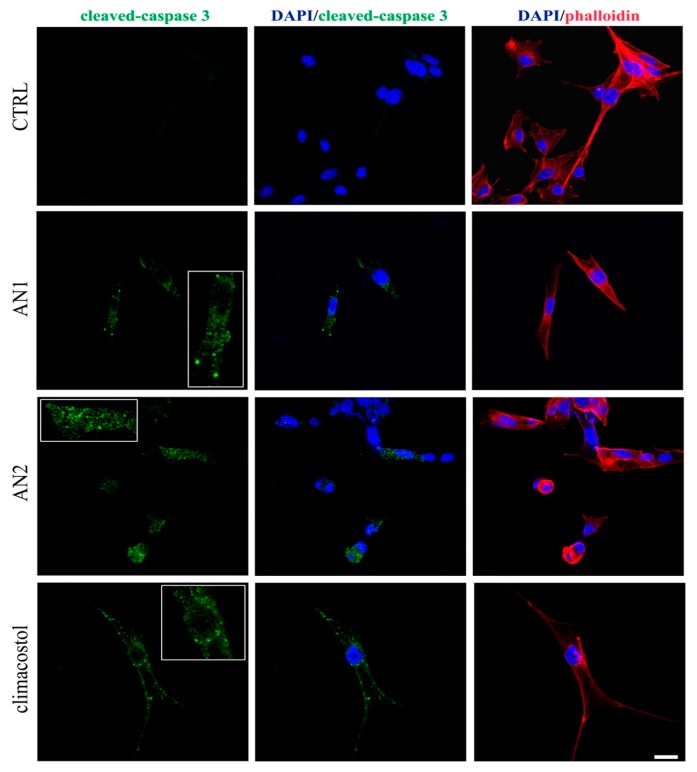
Apoptosis in melanoma cells. Immunofluorescence imaging of cleaved-caspase 3 (punctate green pattern) in B16-F10 cells cultured in the presence of 30 μg/mL AN1, AN2, and climacostol or vehicle (CTRL) for 9 h. DAPI (blue) and phalloidin (red) were used for nuclei and cytoskeleton detection, respectively. The images are representative of six independent experiments. Inserts represent enlarged image details. Scale bar = 20 µm.

The signalling events responsible for the climacostol-induced pro-apoptotic effects in tumours, including melanomas, may couple the activation of the executioner caspase 3 [10,11]. Accordingly, immunostaining with a fluorescently labelled antibody that binds specifically to cleaved (active) caspase 3, a hallmark of apoptosis, revealed that B16-F10 melanoma cells expressed active caspase 3 at 9 h after 30 μg/mL AN1, AN2, and climacostol treatment, whereas no specific stain was observed in vehicle-treated (control) cells (Figure 9). These results demonstrate the pro-apoptotic effects of AN1 and AN2, in agreement with previous results [5,10].

### 2.4. Antimicrobial Activity of Climacostol Analogues

Ethanolic fractions of climacostol, AN1, and AN2 were used in dose–response experiments performed to compare their cytotoxic effects against a panel of bacterial and fungal pathogens, and six species of freshwater ciliates.

The data presented in Table 2 and Table 3 indicate that the two analogues exert an appreciable antimicrobial effect on all the organisms considered in this study, with the exception of *Escherichia coli* and *Pseudomonas aeruginosa* that appear substantially not affected by the activity of the toxins.

Among pathogens, AN1 proved to be the most toxic compound against *S. aureus*, *E. faecalis* (MIC and MBC values of 8 µg/mL) and *C. albicans* (MIC and MBC values of 4 µg/mL).

Similarly, AN1 showed the highest toxicity (0.64 µg/mL < LC_50_ < 2.15 µg/mL) against four ciliates (*B. japonicum*, *P. multimicronucleatum*, *S. ambiguum*, and *S. teres*), both after 1 h and 24 h of treatment, whereas comparable activities were measured for AN1 and climacostol against *E. aediculatus* and *P. tetraurelia* (Table 4).

In contrast to AN1, cytotoxicity values of AN2 appear comparable or worse than those of climacostol, with LC_50_ ranging from 1.17 µg/mL to 4.63 µg/mL.

### 2.5. Induction of Necrosis and Apoptosis in Ciliates

**Figure 10:**
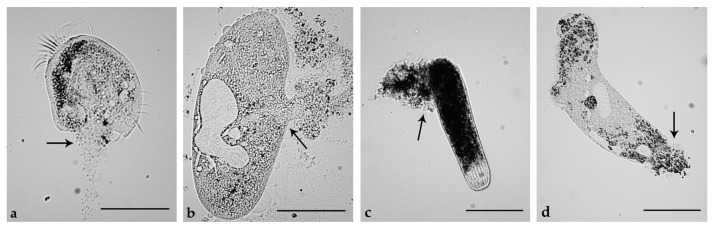
Necrotic effects of AN1 and AN2 toxins on ciliated protists. (**a**) Effects of AN1 on *Euplotes aediculatus* and (**b**) *Paramecium multimicronucleatum*. (**c**) Effects of AN2 on a contracted cell of *Spirostomum ambiguum* and (**d**) *Spirostomum teres*. Cells were exposed to 5 µg/mL solutions of AN1 or AN2 and observed after 20 min. Arrows indicate plasma membrane fractures and products of cell death released into the extracellular space. The images are representative of 10 independent experiments performed for each species. Scale bars = 100 µm.

As previously reported for the effects of climacostol on free-living ciliates [2,5], the cytotoxicity of both AN1 and AN2 on *B. japonicum*, *P. multimicronucleatum*, *P. tetraurelia*, *S. ambiguum*, and *S. teres* appeared to be mediated by necrosis (Table 5), a form of rapid cell injury which does not follow the apoptotic signal transduction pathway. Indeed, ciliates incubated with AN1 or AN2 (see Table 4) were characterized by loss of plasma membrane integrity and uncontrolled release of cellular components into the extracellular space (Figure 10).

On the contrary, whereas AN1 also induced necrosis on *E. aediculatus*, only programmed cell death was triggered on this ciliate by AN2.

**Figure 11:**
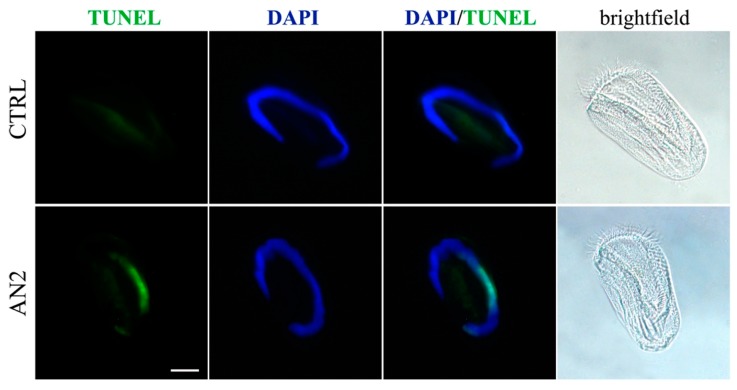
Apoptosis in ciliates. TUNEL assay in *E. aediculatus* treated with 2 μg/mL AN2 or vehicle (CTRL) for 2 h. DAPI (blue) was used for nuclei detection. The images are representative of 10 independent experiments. Scale bar = 30 µm.

In fact, after 1 h of incubation, no apparent change in cell morphology and no sign of necrosis, such as cell swelling and rupture, were visible under light microscopy, even if different cells showed a slowed locomotion and a tendency to remain at the bottom of the depression slide. Within a period of 2 to 6 h of incubation with AN2, the ciliates showed the typical picture of nuclear events associated with apoptosis. In particular, the early steps of nuclear damage were detectable in *Euplotes* by labelling of DNA strand breaks (TUNEL), within 2 h of incubation (Figure 11), whereas no TUNEL stain was detected in the presence of AN1 (data not shown).

**Figure 12:**
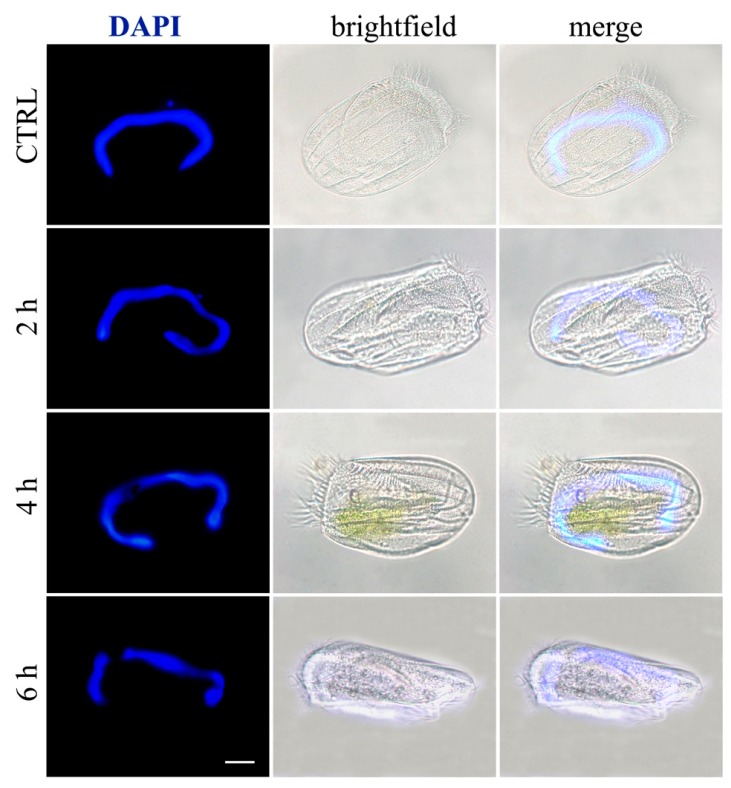
Apoptosis in ciliates. *E. aediculatus* were treated with 2 μg/mL AN2 for increasing times or vehicle (CTRL). DAPI (blue) was used for nuclei detection. The images are representative of 10 independent experiments. Scale bar = 30 µm.

After 4 h of AN2 incubation, DAPI staining showed that the micronucleus disappeared and the macronucleus was starting to fragment (Figure 12). Increased fragmentation of nuclear apparatus was observed after 6 h, confirming the progress of the apoptotic process.

## 3. Discussion

### 3.1. Climacostol and Its Analogues

The alkenylresorcinols are small organic compounds of biogenetic origin via aromatic pathway of interest from pharmacological, biomedical, and biotechnological points of view [13]. A considerable importance in this family of natural products of resorcinolic lipids is given by 1,3-dihydroxybenzene derivatives with 5-alkenyl side chain for their biological qualities [27]. One of the recurrent drawbacks associated with natural products is a limited access to the material. For this reason, chemical synthesis is the best way to solve supply problems, and, in the last years, we have also been involved in studying new synthetic methodologies for small molecules containing a resorcinolic moiety in great quantities in order to carry out the biological tests. Although alkenylresorcinols have a relatively simple structure compared to other derivatives of natural origin, and the preparation schemes require a limited number of synthetic steps, the methodologies studied had to face different problems. The syntheses reported so far in the literature had rather modest yields because of the formation of considerable amounts of by-products structurally similar to the active ingredient, which were therefore difficult to eliminate.

It is known that the role of (Z)-configuration with regard to the hydrocarbon side chain in alkenylresorcinols is crucial in facilitating their biological activity. As a result of our program efforts to develop new synthetic methodologies for agents isolated from natural sources, we studied an innovative diastereoselective synthesis of (Z)-diastereomer alkenyresorcinols with more effectiveness in their biological activity than natural products.

For these reasons, in our studies on alkenylresorcinols that follow the drug likeness Lipinski’s rule [28] such as climacostol and its analogues AN1 and AN2 (Figure 1), we utilized our (Z)-selective methodology reported in Section 2.1.

### 3.2. Mammalian Cells

In this study, we designed and synthesized climacostol analogues AN1 and AN2 carrying an additional methyl group and hydroxyl group, respectively, in the aromatic ring, in an attempt to enhance the anti-cancer and/or antimicrobial activities of the native compound. Both in vitro and in vivo evidence demonstrated that climacostol is a highly effective cytotoxic compound against a wide range of cancer cells, including those affecting humans, activating apoptosis/disrupting autophagy as a general event. This prompted us to propose the protozoan toxin as a potential lead compound for the design of new anti-cancer drugs [1,10,11], also considering that different molecules produced by other protozoan ciliates show some particular pharmacological properties [9]. For example, the sesquiterpenoid euplotin C or the protein pheromone E*r*-1 from the *Euplotes* species, have been previously shown to act respectively as an anti-cancer compound or as an immune-modulatory factor [1,29,30,31,32]; an antibiotic activity against methicillin-resistant *Staphylococcus aureus* has been recognized in the quinone pigment blepharismin isolated from *Blepharisma japonicum* [33].

Our data indicate that the insertion of a methyl or a hydroxyl group in the aromatic ring was not able to augment climacostol activity in mammalian cells since the cytotoxic effects of AN1 and AN2 on a panel of mouse and human cell lines resulted in similar or weaker effects to that of climacostol. Although climacostol was reported to be more toxic against tumours than certain immortalised non-tumour cells [10], here it appeared to be as toxic to C_2_C_12_ as to the tumour cells tested, suggesting that climacostol effects are not necessarily correlated to the cancerous origin of cells. However, the possibility that climacostol preferentially affects cancerous vs. normal (non-transformed non-immortalised) cells remains to be elucidated. From a mechanistic point of view, the compounds carrying the additional groups acted on the death pathway in the same way as the native molecule. Indeed, the ability to induce apoptosis was confirmed for both AN1 and AN2, thus suggesting the same molecular target of climacostol.

### 3.3. Bacteria and Fungi

In contrast to what was observed for mammalian cells, the comparison of the antibiotic activity of climacostol and its analogues against microbial pathogens showed some appreciable differences both in MIC and MBC values obtained for the Gram-positive *S. aureus* ATCC25923 and *E. faecalis* ATCC29212 and for the fungus *C. albicans* ATCC24433. In particular, AN1 resulted in the most active compound against the three microorganisms, whereas AN2 showed markedly weaker activity than that of climacostol against the same pathogens.

According to a previous study performed on the same organisms with climacostol and its synthetic alkynyl and alkyl analogues, AN1 and AN2 appeared to be inactive against the Gram-negative *E. coli* and *P. aeruginosa* [9,14]. As stated by Petrelli et al. [8], it is possible that the lack of effects observed for the climacostol analogues against *E. coli* ATCC25922 and *P. aeruginosa* ATCC27853 can be related to the peculiar structure of the Gram-negative bacterial cell wall, where the outer membrane acts as a selective barrier to prevent the penetration of several compounds, due to the hydrophilic nature of the surface exposed to the environment and to the selectivity of specific outer membrane proteins (i.e., the porins).

The data reported here together with those obtained in previous studies show that the antimicrobial activity of climacostol against bacteria and fungi can be effectively modulated by the derivatization of the aromatic ring with a methyl (i.e., increased effects) or a hydroxyl group (i.e., decreased effects), whereas differences in the saturation rate of the lateral chain, obtained with the synthesis of alkynyl and alkyl analogues, appear unrelated to the activity of the molecule [8].

### 3.4. Ciliates

Some years ago, Buonanno et al. reported that the cytotoxic potential of synthetic alkynyl and alkyl analogues of climacostol on a panel of free-living ciliated protozoa appeared to be inversely correlated to the unsaturation level of their aliphatic chains [6]. More recently, Quassinti et al. [9] observed that the double bond present in the alkenyl chain of the protozoan toxin might facilitate its interaction with both histone-protected and naked DNA, damaging them extensively by inducing appreciable ROS generation, and strongly suggesting that mtDNA could represent a second important target for the toxin.

Here we demonstrate that the derivatization of the aromatic ring of climacostol can also lead to a synthetic compound showing cytotoxic activity against ciliates that is appreciably different from that of the native toxin.

In fact, treatment of *B. japonicum*, *P. multimicronucleatum*, *S. ambiguum*, and *S. teres* with climacostol or AN1 resulted in the lowest LC_50_ values for the analogue, indicating the effectiveness of the addition of a methyl group to the aromatic ring. On the other hand, ciliates treated with climacostol or AN2 did not show relevant differences in the level of cytotoxicity.

Taken together, the data collected in previous and present studies indicate that it is the general structure of the two molecular moieties of climacostol, that is, the hydroxylated aromatic ring and the straight alkenyl chain, which can contribute to the overall cytotoxic behaviour on eukaryotic organisms.

### 3.5. Cell Death in Ciliates

The physiological function of climacostol in natural environment appears to be linked with both the defence against predators and the capability to paralyze prey before ingestion [2,34,35,36]. Similarly, to other offensive/defensive protozoan secondary metabolites characterized to date, the effects of climacostol on prey and predators appear to be essentially mediated by a necrotic process [2,5]. This process refers to a rapid (unprogrammed) cell death, often caused by external factors such as toxins, and it was likely evolutively selected in ciliates to maximize the efficiency of their poisonous compounds on prey and predators (see [36] for a review).

In contrast to climacostol, the analogues AN1 and AN2 used in this study were not selected by evolution as weapons to hunt prey or repel predators, but designed and chemically synthesized in order to explore their structure–activity relationships in different biological models. Therefore, some modifications and/or modulations of the physiological properties of the native compound were expected.

Our data indicate that the cytotoxicity potency of AN1 on ciliates is higher than that induced by climacostol, and that the two compounds appear to share a common mechanism of necrosis to induce cell death.

On the contrary, even if the cytotoxicity level in ciliates treated with AN2 or climacostol are substantially comparable after 24 h, the mechanism of action of the analogue on *E. aediculatus* does not match with a necrotic process, but rather resembles programmed cell death.

In fact, events essentially overlapped with those observed for canonical apoptosis of eukaryotic cells were revealed by fluorescence and light microscopy on *Euplotes* cells incubated with AN2, such as the progressive fragmentation of the macronucleus, the apparent absence of changes in cell morphology, and the lack of severe necrotic damage, such as cell swelling and rupture.

On the one hand, the capability of AN2 to induce apoptosis instead of necrosis in protozoan ciliates is particularly interesting in this context because it confirms the effective possibility to modulate, at least in unicellular eukaryotes, both the toxicity level and the mechanism of action of the lead molecule, by the addition of an OH-radical to the ortho position of the aromatic ring of climacostol. Furthermore, the observation for the first time of apoptosis in *E. aediculatus* extends the number of free-living and parasitic protozoan species where apoptosis-like processes have been reported, confirming that programmed cell death mechanisms have been evolutively conserved in eukaryotes of different complexity, from unicellular to multicellular ones [37,38]. In this regard, even if apoptosis in multicellular eukaryotes is proposed as an adaptive process that contributes to the normal development and functionality of the organism, no definitive or unique explanation is to date available for apoptosis-like processes described for single-celled organisms, thus opening the way to more in-depth investigations on the significance of this “altruistic suicide” in the ancestors of animals. In the future, AN2 may be a good candidate to explore the extension, features, and significance of apoptosis-like processes eventually present in the main groups of free-living ciliates, for one of which a secondary metabolite purified from *Euplotes crassus*, euplotin C, led to a detailed description in *Euplotes vannus* [30].

## 4. Conclusions

The experimental data reported in our study indicate that the structural modification of the aromatic ring of the protozoan toxin climacostol by the addition of a methyl group (to yield AN1) or a hydroxyl group (to yield AN2), resulted in an appreciable variation of both the toxicity and the action mechanism of the native compound. In fact, whereas similar effects were substantially observed for climacostol and its analogues on mammalian cells, AN1 resulted in the most active compound against bacterial and fungal pathogens, and protists.

Finally, the additional hydroxyl group carried by AN2 appears to be the pivotal structural trait that transforms the analogue into an apoptosis-inducing compound in single-celled eukaryotes.

These results bring new clues to the attempt to design and synthetize additional novel analogues of climacostol that can increase or optimize its pharmacological properties.

## 5. Materials and Methods

### 5.1. Cell Cultures

#### 5.1.1. Mammalian Cells

B16-F10 mouse melanoma, GL261 mouse glioma, SK-N-BE human neuroblastoma, CT26 mouse colon cancer, and C_2_C_12_ mouse myoblast cells [39,40,41,42,43] were cultured in Iscove’s, Dulbecco’s Modified Eagle Medium (DMEM) high glucose, or DMEM-F12. Medium was supplemented with 10 and 20% heat-inactivated foetal bovine serum, glutamine (2 mM), penicillin/streptavidin (100 U/mL), and 1% Hepes 1 M (pH 7.4). Cells were grown at 37 °C in a humidified atmosphere containing 5% CO_2_ and routinely passaged every three days at sub-confluent densities.

#### 5.1.2. Bacteria and Fungi

Antimicrobial activity of climacostol and analogues was determined against a panel of microorganisms including *Staphylococcus aureus* ATCC25923, *Escherichia coli* ATCC25922, *Pseudomonas aeruginosa* ATCC27853, *Enterococcus faecalis* ATCC 29212, and *Candida albicans* ATCC 24433.

#### 5.1.3. Ciliated Protists

*Blepharisma japonicum* strain R1072 [35,44], *Euplotes aediculatus* clone EA-III [45,46], *Paramecium multimicronucleatum* clone TL-2 [46], *Paramecium tetraurelia* stock 51 [47], *Spirostomum ambiguum* stock Pol-5 [48], *Spirostomum teres* stock Pol-1 [49], and *Stentor roeseli* clone TL-4 [45] were cultured in synthetic medium for Blepharisma (SMB) (1.5 mM NaCl, 0.05 mM KCl, 0.4 mM CaCl_2_, 0.05 mM MgCl_2_, 0.05 mM MgSO_4_, 2 mM Na-phosphate buffer pH 6.8, 2 × 10^−3^ mM EDTA) and fed with the flagellate *Chlorogonium elongatum*, cultivated as described in [50], or in Jaworski’s Medium (JM) solution.

### 5.2. Climacostol and Its Analogues

Chemically-synthesized climacostol and its two analogues AN1 and AN2 were dissolved in ethanol (1 mg/mL) and stored in the dark at −20 °C. The solutions were diluted with the appropriate medium at the time of the experiment.

### 5.3. 3-(4,5-Dimethylthiazol-2-yl)-2,5-Diphenyltetrazolium Bromide (MTT) Assay

Cell viability was determined by MTT assay using published protocols [29,31,51,52]. Briefly, the assay was performed treating cells for 24 h in the absence (vehicle) or in the presence of increasing concentrations of compounds. MTT absorbance was quantified spectrophotometrically using a Glomax Multi Detection System microplate reader (Promega, Milan, Italy).

EC_50_ (the concentration producing half the maximum effect) and E_max_ concentration (producing the maximum effect) were determined by nonlinear regression curve analysis of the concentration–effect responses. Differences in potency values among concentration–response curves were calculated with the *F*-test. The GraphPad Prism software package (GraphPad Software, San Diego, CA, USA) was used.

### 5.4. Immunofluorescence Detection of Caspase 3 Activity

As previously detailed [10], B16-F10 cells cultured in 120 mm coverslips were fixed in 4% paraformaldehyde in 0.1 M PB, pH 7.4, for 10 min and overnight stained with anti-cleaved-caspase 3 antibody (Cell Signaling Technology, Danvers, MA, USA), in PB containing 0.5% Triton X-100. Cells were then stained with the appropriate Alexa Fluor secondary antibodies (Life Technologies, Monza, Italy), for 1 h and cover-slipped in a ProLong Gold Antifade Mountant (Life Technologies), stained with fluorescein phalloidin (cytoskeleton detection) (Life Technologies) and DAPI (nuclei detection) (Sigma-Aldrich, St. Louis, MO, USA). Slides were analysed using a Zeiss microscope (Axioskop 2 plus, Carl Zeiss, Oberkochen, Germany) equipped with the Axiocam MRC photocamera and the Axiovision software. Images were then optimized for contrast and brightness using Adobe Photoshop (Adobe Systems, Mountain View, CA, USA).

### 5.5. Cytotoxicity Assay on Microorganisms

Climacostol and its analogues were tested against a panel of pathogen microbes including *Staphylococcus aureus* ATCC 25923, *Escherichia coli* ATCC 25922, *Pseudomonas aeruginosa* ATCC 27853, *Enterococcus faecalis* ATCC 29212, and *Candida albicans* ATCC 24433 [53].

Two-fold serial dilutions of each compound (resuspended in ethanol to a final concentration of 10 mg/mL) were prepared in 96-well plates, starting from 512 µg/mL in cation-adjusted Mueller Hinton Broth, to test bacterial strains and RPMI 1640 medium for *Candida albicans*. An equal volume of bacterial (10^6^ colony-forming units (CFU)/mL) or fungal (2.5 × 105 CFU/mL) inoculum was added to each well of the microtiter plate containing 0.1 mL of the serially diluted test molecule. All tests were done in triplicate. After incubation for 18–24 h at 35 °C (24–48 h in the case of *Candida*), in normal atmosphere, the minimum inhibitory concentration (MIC) was determined by the broth microdilution method and the minimum bactericidal concentration (MBC) was subsequently determined as recommended by the European Committee on Antimicrobial Susceptibility Testing guidelines.

To evaluate the toxicity of climacostol and its analogues on ciliates, triplicate samples of 10 ciliate cells were placed in depression slides containing 250 μL SMB and increasing concentrations of each toxin (from 0.5 to 50 μg/mL). The number of surviving organisms (normal morphology and locomotion) was counted, after 1 h and 24 h. The median lethal concentrations (LC_50_) were estimated on the basis of a concentration–survival curve (GraphPad Prism software, version 4.0, GraphPad, San Diego, CA, 2003), and the difference in sample means were expressed using the 95% level of confidence, essentially according to the procedure described in [54].

### 5.6. Necrosis/Apoptosis Detection on Ciliates

The induction of necrosis on ciliates was assessed by light microscopy after 20 min of incubation with climacostol, AN1, or AN2.

For the detection of apoptosis, published protocols were used [29]. Briefly, *E. aediculatus* cells were fixed in paraformaldehyde 4% in PBS, then they were washed in PBS, mounted onto polysin slides, and finally coverslipped with Fluoroshield mounting medium containing DAPI (Abcam, Cambridge, UK). Alternatively, they were processed by TUNEL method (DeadEnd Fluorometric TUNEL System, Promega) according to the manufacturer’s instructions. DAPI staining, fluorescent TUNEL signal, and the corresponding bright field images were acquired using a Zeiss microscope (Axioskop 2 plus, Carl Zeiss, Oberkochen, Germany) equipped with the Axiocam MRC photocamera and the Axiovision software. Images were then optimized for contrast and brightness using Adobe Photoshop (Adobe Systems, Mountain View, CA, USA).

## Figures and Tables

**Table 1:** Parameters of toxin-induced inhibition of cell viability. EC_50_ (µg/mL) = the concentration producing half the maximum effect.

Cell Line	Origin	EC_50_		
		Climacostol	AN1	AN2
B16-F10	mouse melanoma	12.81	18.88	37.94 ^*^
GL261	mouse glioma	28.87	27.32	21.79
SK-NE-BE	human neuroblastoma	19.13	34.31 ^*^	39.84 ^*^
CT26	mouse colon cancer	31.58	36.11	27.42
C_2_C_12_	myoblasts of mouse	16.19	29.28 ^*^	31.72 ^*^

*: *p* < 0.0001 from climacostol value (*F*-test); *n* = 3.

**Table 2:** Antimicrobial activity (minimum inhibitory concentration (MIC)) of climacostol and its analogues against a panel of reference microorganisms.

Compounds	MIC (µg/mL)				
	*S. aureus* ATCC25923	*E. faecalis* ATCC29212	*E. coli* ATCC29212	*P. aeruginosa* ATCC27853	*C. albicans* ATCC24433
**climacostol**	16	16	>128	>128	8
**AN1**	8	8	>128	>128	4
**AN2**	64	128	>128	>128	16

**Table 3:** Minimum bactericidal concentration (MBC) of climacostol and its analogues against a panel of reference microorganisms.

Compounds	MBC (µg/mL)				
	*S. aureus* ATCC25923	*E. faecalis* ATCC29212	*E. coli* ATCC29212	*P. aeruginosa* ATCC27853	*C. albicans* ATCC24433
**climacostol**	16	16	n.d.	n.d.	8
**AN1**	8	8	n.d.	n.d.	4
**AN2**	128	128	n.d.	n.d.	16

**Table 4:** Comparison of the toxic effects of climacostol and its analogues (AN1 and AN2) on various ciliate protists. Viability was assessed after 1 h or 24 h of incubation and the LC_50_ mean values (bold) were obtained by nonlinear regression analysis of three independent experiments with 95% confidence limits calculated using GraphPad Prism 4 software.

Ciliated Protists	Cytotoxicity (LC_50_ µg/mL; 95% C.I.)					
	Climacostol		AN1		AN2	
	1 h	24 h	1 h	24 h	1 h	24 h
*B. japonicum*	**3.17**	**2.04**	**2.15**	**1.55**	**4.63**	**1.94**
	(2.79–3.94)	(1.67–2.48)	(1.82–2.54)	(1.37–1.77)	(3.92–5.47)	(1.81–2.06)
*E. aediculatus*	**1.70**	**0.83**	**1.71**	**0.90**	**2.19**	**1.32**
	(1.62–1.79)	(0.47–1.46)	(1.36–2.16)	(0.25–3.16)	(1.90–2.53)	(0.93–1.87)
*P. multimicronucleatum*	**1.64**	**0.88**	**0.88**	**0.80**	**1.92**	**1.25**
	(0.17–16.22)	(0.18–4.45)	(0.26–3.05)	(0.90–2.59)	(1.69–2.19)	(0.64–2.43)
*P. tetraurelia*	**1.43**	**0.90**	**1.28**	**0.95**	**2.00**	**1.17**
	(0.40–5.06)	(0.37–2.19)	(0.45–3.65)	(0.60–1.50)	(1.79–2.23)	(0.54–2.52)
*S. ambiguum*	**2.03**	**1.66**	**1.46**	**0.64**	**3.43**	**2.04**
	(1.81–2.27)	(1.43–1.92)	(1.16–1.85)	(0.03–15.05)	(2.80–4.19)	(1.65–2.51)
*S. teres*	**2.04**	**1.68**	**1.28**	**0.74**	**3.50**	**1.57**
	(1.10–3.78)	(1.55–1.83)	(0.67–2.44)	(0.03–18.21)	(3.27–3.74)	(1.39–1.86)

**Table 5:** Cytotoxic effects of climacostol, AN1, or AN2 on five species of ciliated protists. N = necrosis; A = programmed cell death.

Ciliated Protists	Effects		
	Climacostol	AN1	AN2
*B. japonicum*	N	N	N
*E. aediculatus*	N	N	A
*P. multimicronucleatum*	N	N	N
*P. tetraurelia*	N	N	N
*S. ambiguum*	N	N	N
*S. teres*	N	N	N
